# Domain interactions of C-terminal Src Kinase determined through NMR spectroscopy with segmental isotope labeling

**DOI:** 10.1007/s13238-016-0333-y

**Published:** 2016-11-04

**Authors:** Dongsheng Liu, Ya Yuan, Rong Xu, David Cowburn

**Affiliations:** 1 iHuman Institute, ShanghaiTech University, 99 Haike Road, Pudong, Shanghai, 201210 China; 2 Department of Biochemistry, Albert Einstein College of Medicine, 1300 Morris Park Avenue, Bronx, NY 10461 USA

**Dear Editor,**

Proteins with large molecular weight exhibit increased number of NMR signals, and the resulting spectral overlap typically imposes limitations. Segmental isotope labeling of individual domains of multidomain protein facilitates spectral interpretation by reducing the number of signals and thus provides a solution to spectral overlap (Liu et al., 2009). This is particularly applicable to multidomain proteins in signaling like kinases. This NMR study was performed using a 50 kDa protein C-terminal Src kinase (Csk) with segmental labeling (Figure S1). The interactions among SH3, SH2 and kinase domains in the solution were identified. Consistent with mutational and structural studies, interdomain interactions were found between the SH2 and kinase domains. On the basis of chemical shift perturbation, the major regions that interacted with the kinase domain included SH3-SH2 and βB-βC linkers, part of βD, βD-βE linker of SH2 domain and SH2-kinase linker region. Thus, phosphotyrosyl ligand binding induced major conformational changes in the SH3-SH2 and βB-βC linkers, part of βD, and βD-βE linker region, thereby allowing modulating kinase activity through domain interactions.

Whether Csk is dimeric or monomeric in solution remains controversial (Ogawa et al., 2002; Levinson et al., 2009; Jamros et al., 2010). The protein sample in the present study was investigated using size exclusion chromatography (SEC), small angle X-ray Scattering (SAXS), dynamic light scattering (DLS) and analytical ultracentrifugation (AUC). Full-length Csk in SEC, SAXS and DLS shows a dimer form and a truncated form of the SH2-kinase protein, which is not dimerized (Figs. S2 and S3) with gel filtration elution, indicating monomer. This result strongly supports the concept that Csk SH3 domain participates in the dimer interface, as suggested by the similarity in the crystal structures of the SH3 dimer (PDB 1CSK) (Ogawa et al., 2002) and SH3 in full-length Csk. The dimerization constant determined via AUC is 1.0 μmol/L (Fig. S3). Analysis of SAXS, DLS, and AUC showed that 3BP1 peptide addition produces a monomeric Csk (Fig. S3), which is presumably formed in vivo in the presence of the prolyl-rich peptide(s) of Lyp phosphatase, as mimicked by 3BP1 in the current experiments.

To perform the segmental labeling of Csk, the selected ligation point is located within the SH2-kinase linker region (V172–E194) that connects the two domains (Fig. S1). Fig. S1C illustrates the generation of the tandem SH3-SH2 (hereafter called as SH32) domain activated at its C terminus as α-thioester and the kinase domain containing a N-terminal cysteine residue. When combined under physiological conditions, the SH32 and kinase domains chemoselectively react through the well-established traditional chemical ligation (Dawson et al., 1994) reaction to form an amide linkage at the ligation junction. Fig. S4A illustrates the purification procedure used in this study, that is, affinity column followed by ion exchange chromatography. MALDI-TOF was used to confirm the molecular weight of the ligation product (Table S1, Fig. S4D and S4E). These results demonstrated that expressed protein ligation methods could be successfully used for segmental labeling of NMR sample in large systems. Fig. 1 shows the overlay of ^1^H-^15^N-HSQC spectra of the Csk SH32 with and without ligated kinase. The red spectrum is isolated Csk [^15^N] SH32, and the blue one is [^15^N] SH32 with ligated unlabeled kinase. Figs S6 and S7 demonstrate the overlay of the ^1^H-^15^N HSQC spectra of [^15^N, ^2^H] kinase with and without ligated SH32 or SH2, respectively.

**Figure 1:**
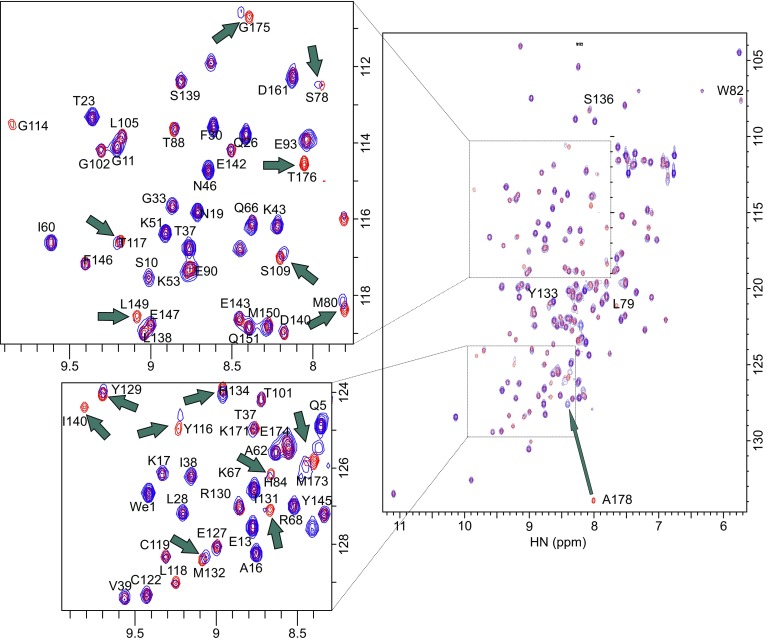
**Structure perturbation of Csk identified by** ^**1**^ **H-** ^**15**^ **N-HSQC spectra**. Overlay of ^1^H-^15^N-HSQC spectra of the Csk SH32 with and without ligated kinase. Red: Csk [^15^N] SH32; Blue: [^15^N] SH32 ligated with unlabeled-kinase. Both of the two samples are concentrated to 0.1 mmol/L and complexed with 1 mmol/L 3BP1. The peaks indicated by arrows show significant chemical shift perturbations

To probe possible minor structural changes among the Csk domains, we measured the chemical shift changes that accompany the formation of full-length Csk through ligation of the kinase domain with the SH32 domain (Fig. 1). Two distinct types of resonance changes were observed in the ^1^H-^15^N HSQC spectrum: (1) chemical shift changes among the peaks, which are indicative of changes in local environment, and (2) resonances that underwent significant line broadening, thus suggesting local changes caused by conformational exchange (Fig. 2A and 2B). These changes are localized to several stretches in the SH2 domain (Fig. 2B). Maximal changes in the chemical shifts were observed for residue A178 (^1^H and ^15^N chemical shifts of 0.41 and 6.38 ppm, respectively) and likely caused by the local effect of ligation. Other changes in chemical shifts occur in four regions, namely, K76–K86 (SH3-SH2 linker), V107-Y116 (βB-βC linker), Y129–S137 (βD, βD-βE linker), and V172-A178 (SH2-kinase linker) regions, are all spatially close to the N-lobe of kinase domain (Fig. 2C) in the full-length Csk crystal structure. The observed shifts indicate that some structural changes in this region accompany the ligation of the kinase domain.

**Figure 2:**
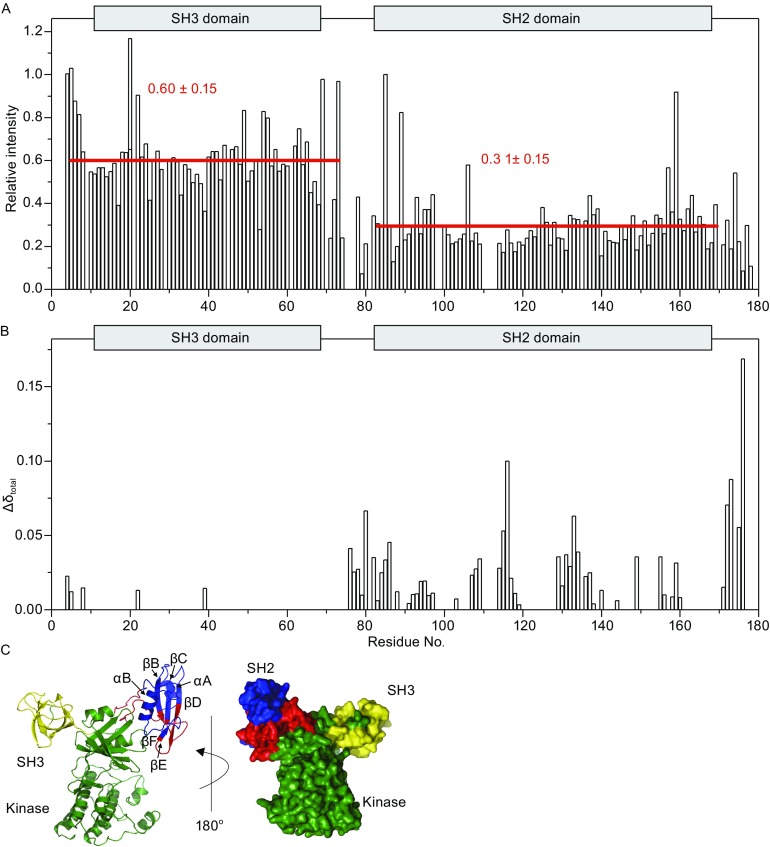
**Structural perturbation observed via segmental labeling methods**. (A) Changes on peak intensity of the SH32 domain with and without ligated kinase domain. The peak intensity of I4 is normalized to 100%. The average relative peak intensity of SH2 (31%) is only half of the SH3 (60%) domain, thereby suggesting a different interaction mode for these two domains. The locations of the SH3 and SH2 domains are indicated above the plot. (B) The combined chemical shift change of a particular residue upon ligation with the kinase domain. (C) Chemical shift perturbation mapped onto the crystal structure of Csk (1K9A **-** A). The red regions are the regions that undergo a big chemical shift change before and after SH32 ligation with the kinase domain

The ligation of the kinase domain to the SH32 domains causes a global reduction of signal intensities because of the increase in the molecular weight of the ligated SH32-kinase protein. Consequently, the overall correlation time is increased. A close inspection shows differential line broadening, which reveals the different dynamic interactions between SH3 and SH2 with the kinase domain. As shown in Fig. 2A, the average relative peak intensity of SH2 (30%) is only half of that of the SH3 (60%) domain (normalized against the peak intensity for residue I4), suggesting that conformational changes occur in the SH2 domain in the solution. This observation is consistent with the crystal structure containing dual SH2 conformations, which causes active and inactive kinase status. Additionally, this phenomenon can occur either because of a tight interaction of the SH2 domain with the kinase domain or from the conformational exchange between states.

Superposition of the ^1^H-^15^N HSQC spectra of free SH2 and kinase ligated SH2 shows the chemical shift perturbations upon ligation (Fig. S5A). The largest of the observed chemical shift perturbations occurred at S78, L79, and M80, and the C-terminal tail of the SH2 domain, with the largest absolute change involving Y116. From the crystal structure of kinase, we can hypothesize that this arises from the close hydrophobic interaction among the SH2-kinase linker and the βBC, βC and two β-strands on kinase domain (Fig. S5B). These data show that interaction between the SH2 and kinase domain described above is present even in the absence of the SH3 domain.

While chemical shift perturbations can be a useful means of mapping interfacial regions within Csk, they are not commonly indicative of the degree of conformational plasticity involved. This structure is considerably different from the active and inactive molecules in the SH2-kinase interface in the full-length Csk. The interactions between the βD-βE loop of the SH2 domain and β3-βC loop in the N-lobe of kinase domain are absent in putative inactive molecules (Ogawa et al., 2002). The comparison of SH2 structures in active full length, inactive full length, and isolated form also shows that the βD-βE and βB-βC loop positions are significantly changed between the active form, inactive form and isolated SH2 (Liu and Cowburn, 2016). βD-βE and βB-βC loops in active form of SH2 from full-length Csk show major domain interactions, and inactive form SH2 from full-length Csk shows minor domain interactions.

Ligands for the SH3 and SH2 domains may play significant roles, in controlling the kinase activities. Cbp binding potentially activates ligand localization for Csk kinase activity (Takeuchi et al., 2000; Wong et al., 2005). Csk and the cytoplasmic domain of phosphorylated Cbp form a stable complex and enhance the affinity of Csk toward Src (Takeuchi et al., 2000). Csk binding to Cbp increases the catalytic activity of the former, as identified *in vitro* and *in vivo*. This activation is accompanied by a two- to six- fold decrease in the *K*_*m*_ for Src in the presence of Cbp or Cbp peptide (Takeuchi et al., 2000). Csk could be regulated by coupling of the SH2 and kinase domains; this coupling may provide an intramolecular activation mechanism for Csk (Ogawa et al., 2002).

In addition to the SH3-SH2 linker, three other identified regions, namely, βB-βC linker, part of βD and βD- βE linker, and the SH2-kinase linker, underwent changes in chemical shift upon ligation with kinase domain; these regions are all spatially close to the kinase domain N-lobe in the crystal structure. One of the largest observed chemical shift perturbations occurred for residue Y116 (0.01 and 0.40 for ^1^H and ^15^N, respectively). The Y116A mutant lost its ability to be activated by SH2 domain ligand almost completely, which suggests that the hydrophobic pocket formed by the side chains of Y116, Y133, L138 and L149 must be intact for the SH2 domain-mediated activation of Csk (Mikkola and Gahmberg, 2010). Notably, in the presence of the SH3 domain, the SH2 domain is structurally close to the Csk kinase domain, thereby causing further structural interactions in the βB-βC linker, part of the βD and βD-βE linkers, and SH2-kinase linker with kinase domain. This result can be explained by the fact that only the SH3-SH2 linker region still retains a significant interaction with the kinase domain on SH3 truncation. The ligation of kinase domain to the SH32 domain causes a global change of signal intensities, which shows that ligation increases the overall correlation time, as expected. However, the intensity variations are not uniform across resonances from both the SH3 and SH2 domains, thus suggesting a different interaction mode for these two domains and partially confirming a tight association of the SH2 domain with the kinase domain.

The ligand to Csk SH2 is Cbp. Cbp has been implicated in various aspects of cancer cell biology (Hrdinka and Horejsi, 2014). Cbp could function as both a positive and negative regulator of mast cell signaling, depending upon the signaling pathway involved (Draberova et al., 2014). Cbp also serves as a central inhibitor of c-Src and other Src family kinases, as a novel tumor suppressor in neuroblastoma (Agarwal et al., 2016). Previously, we have studied the interaction of Csk L-SH2 with Cbp peptide using L-SH2 sample with and without binding with Cbp peptide (Liu and Cowburn, 2016). The perturbed residues upon Cbp binding indicate that βB-βC, βD and βD-βE loops of L-SH2 undergo significant conformational change. In addition, βA-βA loop residues at the N-terminal also undergo small-range perturbation upon the binding of phosphopeptide with K86 and I87 in this loop. These results suggest that Cbp binding causes dramatic conformational changes in the βB-βC, βD and βD-βE loops, which are all spatially close to the N-lobe of Csk kinase domain. In the present study, we focused on how the activation signal is transferred from the peptide-bound SH2 domain to the kinase domain using segmental labeling NMR methods. Comparison of phosphotyrosyl peptide-bound Src SH2 domain crystal structure (1SPS) with free Csk SH2 (with SH3-SH2 linker) crystal structure revealed that the βB- βC and βD-βE loops of the SH2 domain exhibit the largest conformational change upon complex formation. In addition to the Y116, which is the largest perturbation found in NMR, the Y112 conformation is changed significantly with and without the presence of kinase domain. In the lack of proper interacting partner, Y112 side chain folds back to SH2 domain. Overall, we suggest that phosphotyrosyl ligand binding causes conformational changes in the SH3-SH2 and βB- βC linkers, part of the βD, and βD-βE linker region, thereby allowing for modulation of kinase activity.

## Electronic supplementary material

Below is the link to the electronic supplementary material.

Supplementary material 1 (PDF 3560 kb)
