# Supplementary material for: Domain interactions of C-terminal Src Kinase determined through NMR spectroscopy with segmental isotope labeling
Source: Protein Cell. 2016 Nov 4;8(1):67–71. doi: 10.1007/s13238-016-0333-y (PMC5233614; doi:10.1007/s13238-016-0333-y)
Supplement: Supplementary file 1 — Supplementary material 1 (PDF 3560 kb) [file 13238_2016_333_MOESM1_ESM.pdf]

Materials and methods

Cloning, expression, and purification

The genes for the SH32 and SH2 domain, which contain residues M1-A178 or A73-A178, respectively, was cloned into the expression vector pTWIN1. The vectors were named pTWIN1-SH32 and pTWIN1-SH2, and the corresponding protein products were named SH32-intein2-CBD and SH2-intein2-CBD, respectively. The Csk kinase domain gene containing residues A179-L450 with an A179C mutation and C-terminal His-tag was amplified and cloned into the expression vector pTWIN1. The plasmid was named pTWIN1-kinase-His, and the designed fusion protein was named CBD-intein1-kinase-His. Overexpression and purification of proteins were described elsewhere (Liu et al., 2009). The protein tyrosine phosphatase PEP proline-rich peptide (PEP-3BP1, residues 605-629, SRRTDDEIPPPLPERTPESFIVVEE) was expressed and purified as previously described (Ghose et al., 2001).

Segmental labeling of NMR sample

Kinase protein mixture was added to the chitin column bound with SH32-intein2-CBD with Buffer A (50 mM Tris-HCl, pH 7.5, 200 mM NaCl). The molar ratio of SH32 or SH2 α-thioester and N-terminal cysteine kinase was 1:1. The system was added with 0.5 mM EDTA and 200 mM 2-mercapto-ethanesulfonic acid. Subsequently, the column was stored at 15 °C for 72 h. During storage, three reactions proceeded: thiolysis of SH32-intein2-CBD to form SH32 α-thioester, degradation of fusion CBD-intein1-kinase-His to form kinase-His, and reaction of SH32 α-thioester with kinase-His with the N-terminal

cysteine (Figure 1C). On-column ligation increases the ligation efficiency (Vitali et al., 2006).

Ligation mixture was eluted from the chitin column and dialyzed against buffer A at 4 °C. The solution was loaded on 10 ml of TALON beads charged with Co^{2+} and pre-equilibrated with buffer A. The column was washed with 50 ml of buffer A to remove the unbound protein (mainly inteins, SH32, or SH2; Figures 2A and 2B). PEP-3BP1 is used to reduce the self-association of the SH3 domain (Borchert et al., 1994; Ghose et al., 2001). The column was washed with another 10 ml of buffer A with 1 mM PEP-3BP1 peptide to remove the SH32 domain bound with the ligation product because of the dimerization of SH3 domain. The bound protein was eluted and further purified by using ion exchange chromatography.

NMR spectroscopy

All NMR experiments were performed at 25 °C on Bruker 900 MHz, 800 MHz, or 700 MHz spectrometers equipped with triple-resonance cryoprobes. Protein solutions were prepared in the following buffer conditions: 0.5–1.0 mM protein in 50 mM Tris-HCl (pH 7.5), 10 mM DTT, 1.0 mM EDTA, 0.01% (w/v) NaN_3, 5% D_2O, and 0.1 mM DSS (4,4-dimethyl-4-silapentane-1-sulfonate). For the experiments containing SH32, a solution of 3BP1 peptide dissolved in NMR buffer was titrated into a SH32 or SH32-kinase-His solution until the peptide was in a molar ratio of 1:2. All the NMR samples were sealed in the NMR tube under nitrogen atmosphere. 3D triple-resonance experiments, namely, HNCA, HN(CO)CA, HNCO, HN(CA)CO, HNCACB and CBCA(CO)NH were performed to

determine backbone resonance assignments. ^1H chemical shifts were referenced to internal DSS. ^{13}C and ^{15}N chemical shifts were referenced indirectly using the $^1H/^{13}C$ or $^1H/^{15}N$ frequency ratios of the zero point 0.101329118 (^{15}N) and 0.251449530 (^{13}C) (Live et al., 1984; Wishart et al., 1995). The combined chemical shift change of a particular residue upon ligation with the kinase domain was calculated as: $\Delta\delta_{total}=sqrt[(\Delta\delta_H)^2+(0.154\ \Delta\delta_N)^2]$ (Mulder et al., 1999). $\Delta\delta_H$ and $\Delta\delta_N$ correspond to the amide proton and nitrogen chemical shift changes upon kinase binding to SH32 or SH2, respectively. The weight factor for ^1H and ^{15}N was determined from the ratio of the average variances of amide nitrogen and proton chemical shifts observed for the 20 common amino acid residues in proteins as deposited with the BioMagResBank. The NMR assignments of the SH32 domain of Csk were deposited to BMRB with the accession number 16017.

Small-Angle X-Ray Scattering Experiments.

The small-angle X-ray scattering (SAXS) data for Csk with and without 3BP1 peptide were collected using the X9 beamline at Brookhaven National Laboratory. The purified proteins were stored in a buffer containing 50 mM Tris-HCl (pH 7.5), 10 mM DTT, 1.0 mM EDTA. Each blank or sample was measured in triplicate, and the sample measurements were adjusted by subtracting the scattering from buffer-alone.

Accession numbers

The NMR assignments of the SH32 domain of Csk were deposited to BMRB with the accession number 16017.

Figure S1

Function, structure and segmental labeling procedure of Csk. (A) Regulation of SFKs by Csk through Cbp binding in lipid rafts. (B)The monomeric structure of Csk with bound 3BP1 (to SH3) and CBP peptide (to SH2). The overall structure is plotted with active Csk 1K9A-A. The position of PEP-3BP1 and CBP peptide is modeled according to the structure of 1JEG and 1SPS, respectively. (C) Schematic depiction of the segmental labeling procedure of human Csk protein.

Figure S2

Size exclusion chromatography and small angle X-ray scattering of Csk samples. Protein solutions are prepared in conditions: 0.5–1.0 mM protein in 50 mM Tris-HCl (pH 7.5).

Figure S3

Sedimentation equilibrium analysis of Csk: The plot shows the concentration (Y-axis) depending on the radius (cm), which is the distance to the rotor in the center. All graphs show the experimental data with their fit and the plots for the sedimentation speed at 9,000 rpm. The experimental data collected at 15,000 rpm are shown by empty dots. The panel on the upper left shows the aberration of the experimental data from the fit

using the monomer–dimer equilibrium model. (A) Full-length Csk alone. (B) Full-length Csk in the presence of 3BP1. (C)The molecular weight (kDa) of Csk calculated from the AU experimental results using different models are shown for different models and dissociation constant for full-length Csk.

Figure S4

Protein ligation, purification and chariazation of SH32-kinase or SH2-kinase. (A)Procedure used for the purification of the SH32-kinase ligation products. (B) Expression and purification of CBD-intein1-kinase-His (lanes 1, 2, 3, and 4) and SH32-intein2-CBD (lanes 5, 6, 7, and 8). Lanes 1 and 2, insoluble and soluble fractions of cell lysate; lanes 3 and 4, flow through and elution of TALON beads; lanes 5 and 6, insoluble and soluble fractions of cell lysate; lanes 7 and 8, flow through and elution of chitin beads. (C). Ligation and purification of SH2-kinase-His. Lanes 1 and 2, CBD-intein1-kinase-His before and after TALON bead purification; lane 3, flow through of chitin beads; lane 4, on-column cleavage and ligation of SH2-kinase-His; lane 5, elution of ligation product from chitin beads; lanes 6 and 7, flow through and elution of TALON beads; lane 8, molecular weight ladder. (D), (E) Selected mass results of the ligation product (Table S1).

Figure S5

Structural perturbation of SH2 domain observed via segmental labeling methods. (A) Overlay of ^{1}H-^{15}N HSQC spectra of the Csk SH2 with and without ligated kinase. Red: ^{15}N-SH2; blue: ^{15}N-SH2 ligated with unlabeled kinase. Both of the two samples are

concentrated to 0.1 mM. The residues that undergo significant chemical shift changes are indicated by arrow on the spectrum. (B) Magnified view of the binding pocket in the SH3-SH2 and SH2-kinase linker regions.

Figure S6

Structure perturbation of Csk identified by ^{1}H-^{15}N-HSQC spectra. Overlay of ^{1}H-^{15}N-HSQC spectra of the $^{15}N^{2}H$ kinase with and without ligated SH32. Red: Csk $^{15}N^{2}H$-labeled kinase; Green: $^{15}N^{2}H$-labeled kinase ligated with unlabeled SH32. The peaks indicated by arrows show significant chemical shift perturbations.

Figure S7

Structure perturbation of Csk identified by ^{1}H-^{15}N-HSQC spectra. Overlay of ^{1}H-^{15}N-HSQC spectra of the $^{15}N^{2}H$ kinase with and without ligated SH2. Red: Csk $^{15}N^{2}H$-labeled kinase; Green: $^{15}N^{2}H$-labeled kinase ligated with unlabeled SH2. The peaks indicated by arrows show significant chemical shift perturbations.

Figure S1

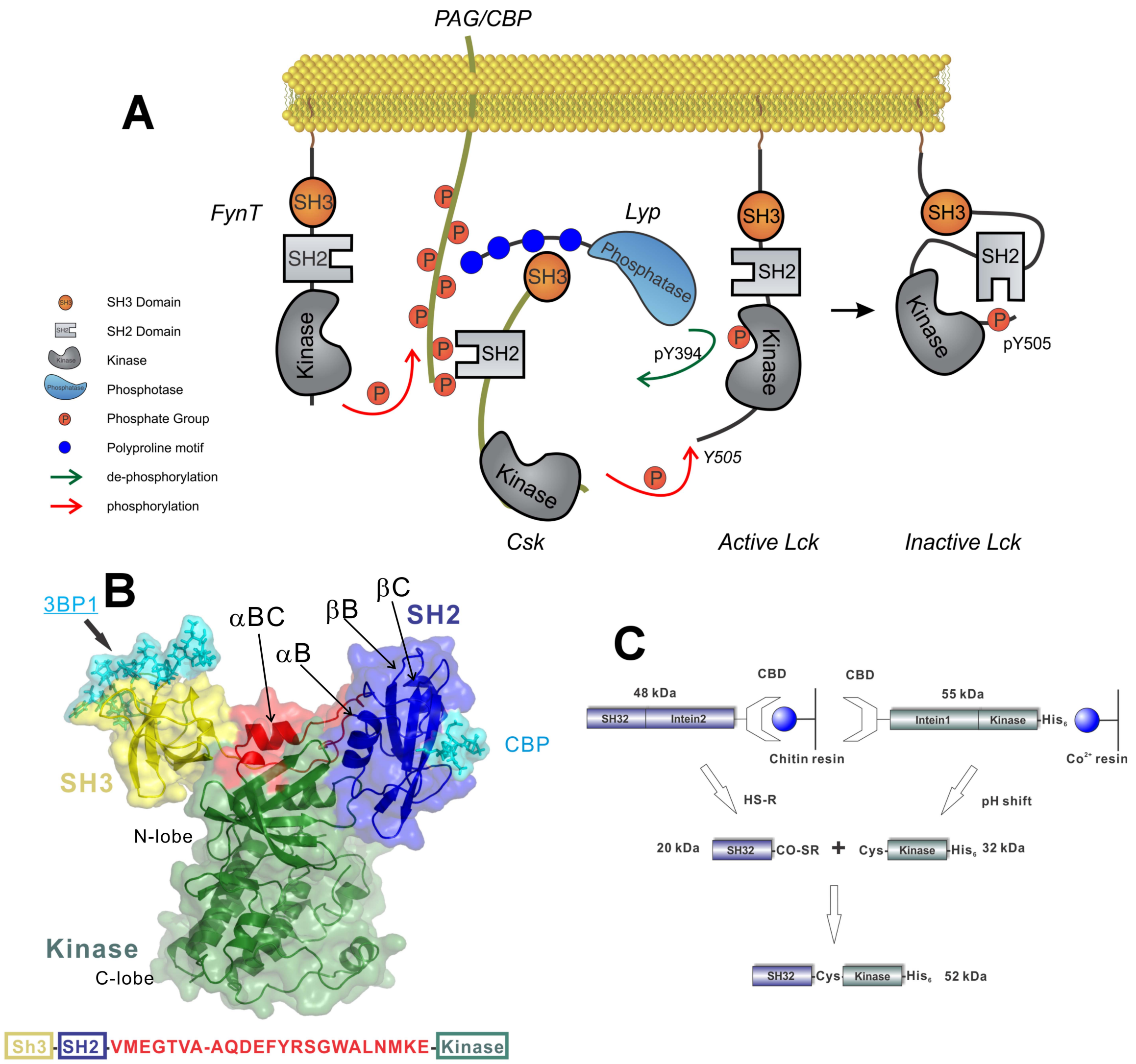
A
PAG/CBP
FynT
SH3
SH2
Kinase
Lyp
Phosphatase
pY394
Y505
Csk
Active Lck
Inactive Lck
pY505
SH3 Domain
SH2 Domain
Kinase
Phosphotase
Phosphate Group
Polyproline motif
de-phosphorylation
phosphorylation
B
3BP1
αBC
αB
βB
βC
SH2
CBP
SH3
N-lobe
Kinase
C-lobe
Sh3-SH2-VMEGTVA-AQDEFYRSGWALNMKE-Kinase
C
48 kDa
SH32
Intein2
CBD
Chitin resin
55 kDa
Intein1
Kinase
His6
Co2+ resin
HS-R
pH shift
20 kDa
SH32-CO-SR + Cys-Kinase-His6 32 kDa
SH32-Cys-Kinase-His6 52 kDa

Figure S2

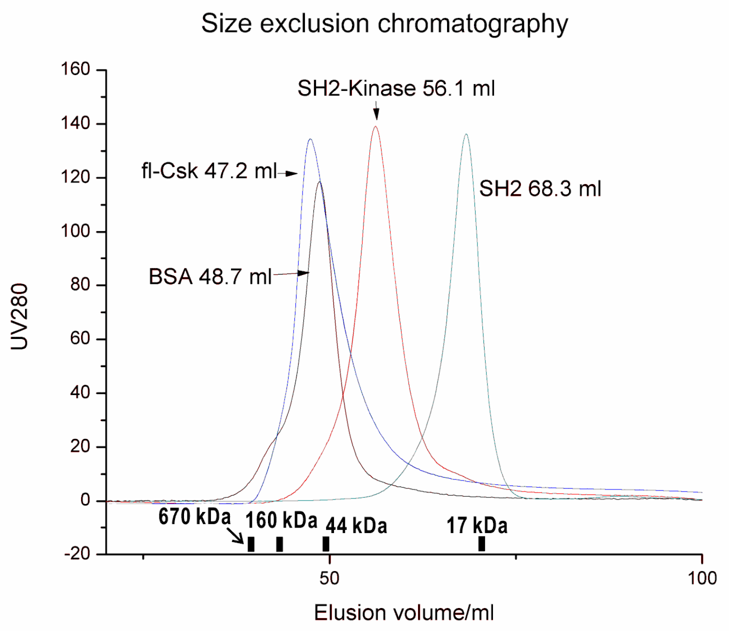
Size exclusion chromatography
SH2-Kinase 56.1 ml
fl-Csk 47.2 ml
SH2 68.3 ml
BSA 48.7 ml
670 kDa
160 kDa
44 kDa
17 kDa
UV280
Elusion volume/ml
160
140
120
100
80
60
40
20
0
-20
50
100

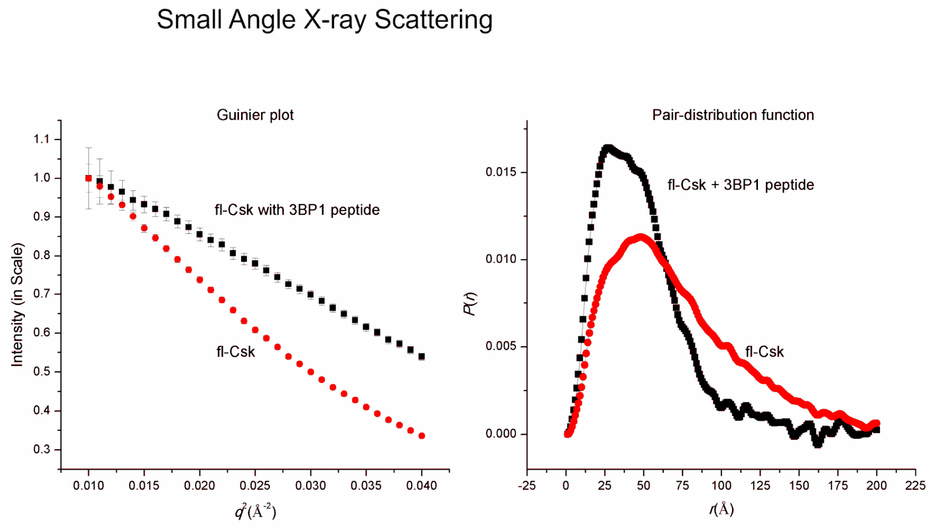
Small Angle X-ray Scattering
Guinier plot
fl-Csk with 3BP1 peptide
fl-Csk
Intensity (in Scale)
1.1
1.0
0.9
0.8
0.7
0.6
0.5
0.4
0.3
0.010
0.015
0.020
0.025
0.030
0.035
0.040
q^2(Å^{-2})
Pair-distribution function
fl-Csk + 3BP1 peptide
fl-Csk
$P(r)$
0.015
0.010
0.005
0.000
-25
0
25
50
75
100
125
150
175
200
225
r(Å)

Figure S3

(A)

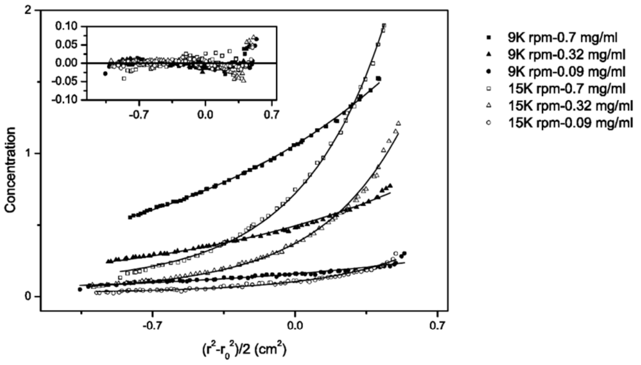

(B)

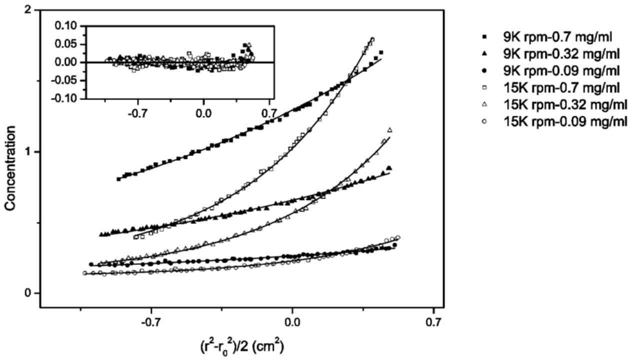

(C)

	Ideal Model	Monomer-Nmer	Dissociation constant
Fulllength Csk	93kD	50kD	1 uM
Fulllength Csk + 3BP1 peptide	63kD		

Figure S4

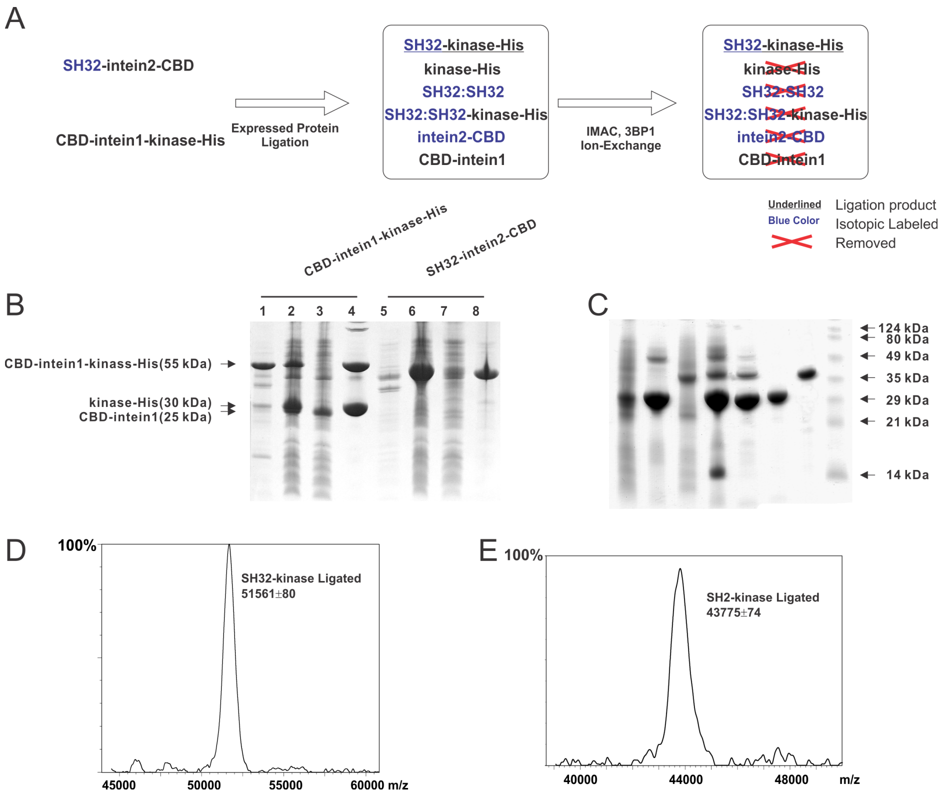

A
SH32-intein2-CBD
CBD-intein1-kinase-His
Expressed Protein Ligation
SH32-kinase-His
kinase-His
SH32:SH32
SH32:SH32-kinase-His
intein2-CBD
CBD-intein1
IMAC, 3BP1 Ion-Exchange
Underlined Ligation product
Blue Color Isotopic Labeled
Removed
CBD-intein1-kinase-His
SH32-intein2-CBD
B
1 2 3 4 5 6 7 8
CBD-intein1-kinass-His(55 kDa)
kinase-His(30 kDa)
CBD-intein1(25 kDa)
C
124 kDa
80 kDa
49 kDa
35 kDa
29 kDa
21 kDa
14 kDa
D
100%
SH32-kinase Ligated
51561±80
45000
50000
55000
60000 m/z
E
100%
SH2-kinase Ligated
43775±74
40000
44000
48000
m/z

Figure S5

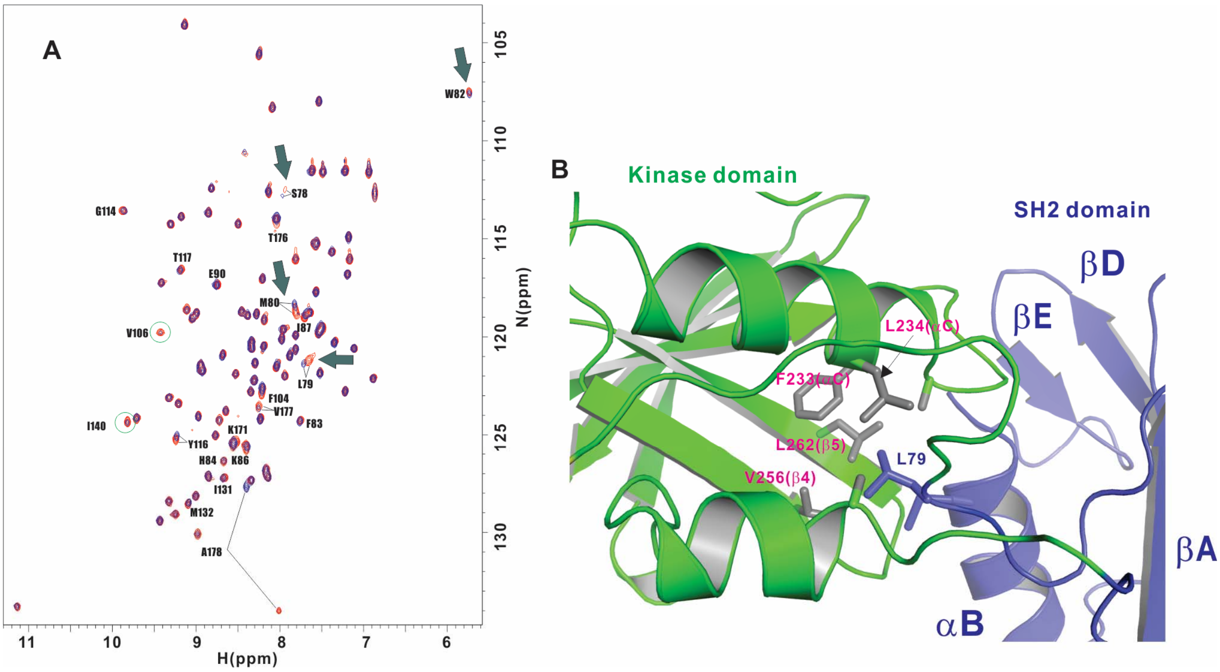

A
W82
S78
G114
T176
T117
E90
M80
V106
I87
L79
F104
V177
F83
I140
K171
Y116
H84
K86
I131
M132
A178
H(ppm)
N(ppm)
B
Kinase domain
SH2 domain
βD
βE
L234(αC)
F233(αC)
L262(β5)
L79
V256(β4)
βA
αB

Figure S6

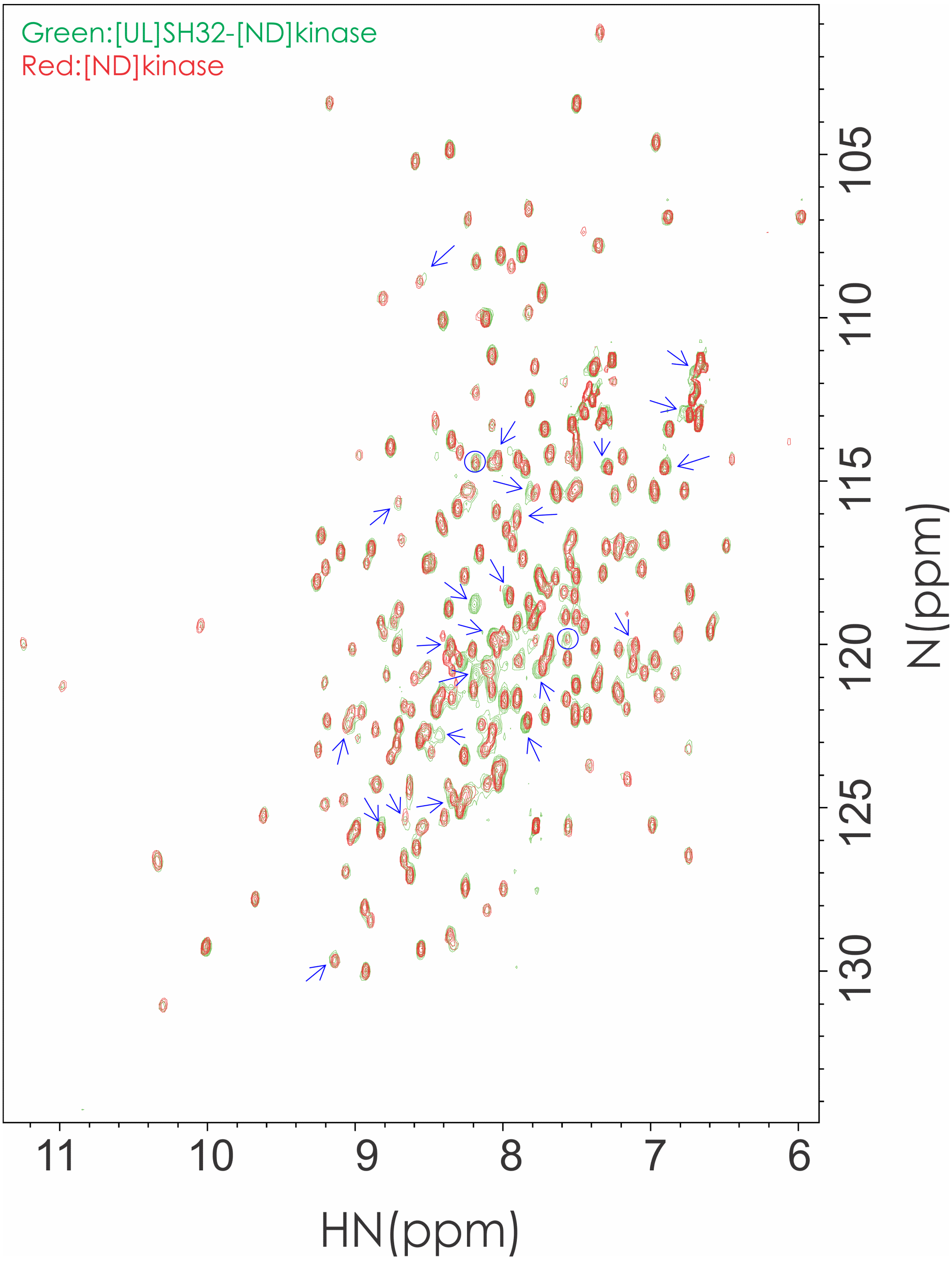
Green:[UL]SH32-[ND]kinase
Red:[ND]kinase
105
110
115
120
125
130
N(ppm)
11
10
9
8
7
6
HN(ppm)

Figure S7

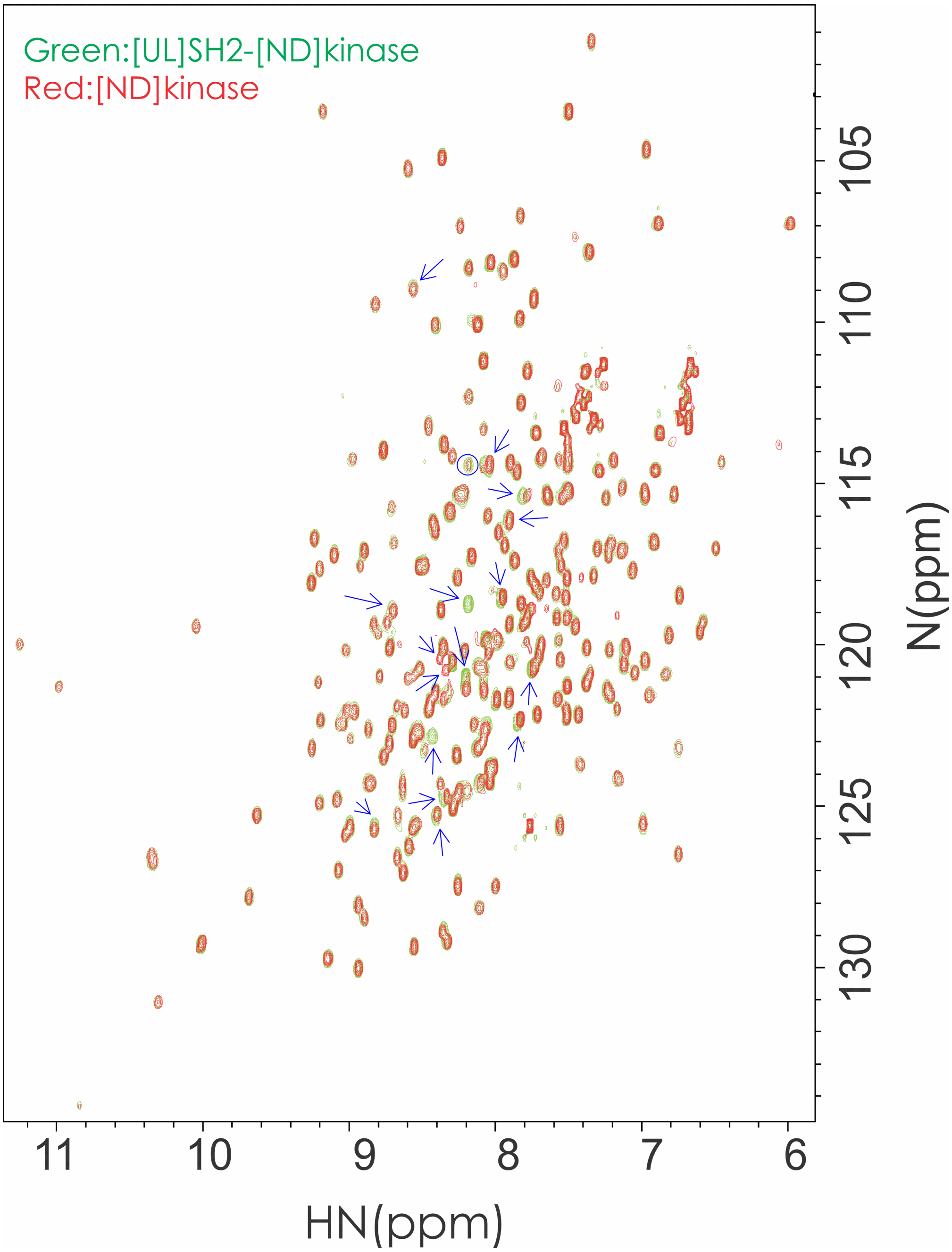

Green:[UL]SH2-[ND]kinase
Red:[ND]kinase
105
110
115
120
125
130
N(ppm)
11
10
9
8
7
6
HN(ppm)

Table S1. Calculated and observed mass. All the calculations are based on natural abundance.

Sample	Theoretical	Observed
SH32	20082	20072±52
SH2	12278	12287±25
Kinase-His	31491	31516±36
SH32-kinase-His (Ligated)	51555	51561±80
SH2-kinase-His (Ligated)	43751	43775±74
SH32:SH32-kinase-His (Dimer)	71637	71651±97
SH32:SH32 (Dimer)	40165	40192±82
Intein2-CBD	27856	27850±30
CBD-intein1	25179	25171±13

References:

Borchert, T.V., Mathieu, M., Zeelen, J.P., Courtneidge, S.A., and Wierenga, R.K. (1994). The crystal structure of human CskSH3: structural diversity near the RT-Src and n-Src loop. FEBS Lett 341, 79-85.

Ghose, R., Shekhtman, A., Goger, M.J., Ji, H., and Cowburn, D. (2001). A novel, specific interaction involving the Csk SH3 domain and its natural ligand. Nat Struct Biol 8, 998-1004.

Liu, D., Xu, R., and Cowburn, D. (2009). Segmental isotopic labeling of proteins for nuclear magnetic resonance. Methods Enzymol 462, 151-175.

Live, D.H., Davis, D.G., Agosta, W.C., and Cowburn, D. (1984). Long-Range Hydrogen-Bond Mediated Effects in Peptides - N-15 Nmr-Study of Gramicidin-S in Water and Organic-Solvents. Journal Of The American Chemical Society 106, 1939-1941.

Mulder, F.A., Schipper, D., Bott, R., and Boelens, R. (1999). Altered flexibility in the substrate-binding site of related native and engineered high-alkaline Bacillus subtilisins. J Mol Biol 292, 111-123.

Vitali, F., Henning, A., Oberstrass, F.C., Hargous, Y., Auweter, S.D., Erat, M., and Allain, F.H.T. (2006). Structure of the two most C-terminal RNA recognition motifs of PTB using segmental isotope labeling. EMBO Journal 25, 150-162.

Wishart, D.S., Bigam, C.G., Yao, J., Abildgaard, F., Dyson, H.J., Oldfield, E., Markley, J.L., and Sykes, B.D. (1995). 1H, 13C and 15N chemical shift referencing in biomolecular NMR. J Biomol NMR 6, 135-140.
